# Recent Advances in Robotic Surgery for Urologic Tumors

**DOI:** 10.3390/medicina60101573

**Published:** 2024-09-25

**Authors:** Sen-Yuan Hong, Bao-Long Qin

**Affiliations:** Department of Urology, Tongji Hospital, Tongji Medical College, Huazhong University of Science and Technology, Wuhan 430030, China

**Keywords:** robotic surgery, Da Vinci SP system, 3D reconstruction, augmented reality, artificial intelligence

## Abstract

This review discusses recent advances in robotic surgery for urologic tumors, focusing on three key areas: robotic systems, assistive technologies, and artificial intelligence. The Da Vinci SP system has enhanced the minimally invasive nature of robotic surgeries, while the Senhance system offers advantages such as tactile feedback and eye-tracking capabilities. Technologies like 3D reconstruction combined with augmented reality and fluorescence imaging aid surgeons in precisely identifying the anatomical relationships between tumors and surrounding structures, improving surgical efficiency and outcomes. Additionally, the development of artificial intelligence lays the groundwork for automated robotics. As these technologies continue to evolve, we are entering an era of minimally invasive, precise, and intelligent robotic surgery.

## 1. Introduction

With the rapid development of computer technology, surgical robots, which integrate medicine, mechanics, and computer science, have emerged. In 2000, Intuitive Surgical’s Da Vinci robot became the first to receive FDA approval for laparoscopic surgery, sparking a revolution in minimally invasive procedures. Initially applied in urology, the Da Vinci surgical robot has demonstrated significant advantages, particularly in surgeries for urologic tumors. Robotic surgery enables precise suturing during partial nephrectomy through a high-definition imaging system and flexible robotic arms. It also facilitates delicate nerve-sparing and lymph node dissection during radical prostatectomy, enhancing patients’ urinary and sexual function outcomes. As the use of robotic surgery in urologic oncology continues to expand, advancements in robotic systems and associated technologies are increasingly focused on achieving minimally invasive, precise, and intelligent procedures. This narrative review was performed on the Pubmed database using the following search strategy: ((((robot-assisted radical prostatectomy) OR (robot-assisted radical cystectomy)) OR (robot-assisted partial nephrectomy)) OR (robot-assisted radical nephrectomy)) AND ((((((da Vinci single-port) OR (Senhance)) OR (three-dimensional reconstruction)) OR (augmented reality)) OR (indocyanine green)) OR (artificial intelligence)). Representative studies about recent developments in robotic surgery for urologic tumors were selected and reviewed.

## 2. Advances in Robotic Surgery Systems

### 2.1. Da Vinci Single-Port (SP) System

Laparoendoscopic single-site surgery (LESS) is a significant milestone in the evolution of minimally invasive surgery, reducing the number of incisions required for surgical procedures to just one [1]. Compared to traditional multi-port laparoscopic surgery, LESS offers advantages such as reduced trauma, less pain, and improved cosmetic outcomes. However, LESS has limitations, including the absence of triangulation and restricted operating space, which can extend surgery time and compromise safety. Robot-assisted laparoendoscopic single-site surgery (RA-LESS) addresses some of these limitations by utilizing the Da Vinci multi-port systems (e.g., Si, Xi) to operate flexible instruments through a single skin incision [2]. Despite this advancement, instrument collision remains a challenge. To overcome this, the latest Da Vinci SP system was developed and received FDA approval for urologic surgery on 31 May 2018. The SP system, a true single-port surgical robot, integrates three multi-jointed instruments and a fully articulating 3D high-definition endoscope within a single robotic arm [3]. This arm enters the patient’s body through a 25 mm incision, where the instruments, designed with “elbow” joints, spread apart to establish optimal triangulation, reducing instrument collision and enabling precise surgical operations.

SP robot-assisted radical prostatectomy (SP-RARP) has been implemented and reported across multiple centers. Agarwal and colleagues first presented data on SP-RARP at the European Urology, where three experienced surgeons performed 49 SP-RARP procedures. The average operative time was 161 min, with a mean blood loss of 200 mL, an 8.1% complication rate (all Clavien grade II), and a mean postoperative hospital stay of one day [4]. A recent meta-analysis of seven studies involving 1239 patients compared the outcomes of the Da Vinci SP system and the multi-port systems (Si, Xi) [5]. The study found no statistically significant differences in operative time, blood loss, continence rates, postoperative complications, positive surgical margins, or biochemical recurrence. However, the SP group had significantly shorter hospital stays and catheterization times, and lower opioid use, indicating less trauma and faster recovery. Thus, SP-RARP is a safe and feasible surgical option.

SP robot-assisted radical cystectomy (SP-RARC) has also shown favorable clinical outcomes. Kaouk et al. first reported the successful implementation of SP-RARC with ileal conduit urinary diversion and shared their initial experience [6]. Gross et al. compared the efficacy of the Da Vinci SP and multi-port systems and found no significant differences in operative time, blood loss, postoperative complications, or positive margins [7]. However, the SP group had a lower number of lymph nodes retrieved, suggesting a potential advantage of SP-RARC in specific contexts. Though limited by a small sample size, this study indicates the potential superiority of SP-RARC. Research on SP robot-assisted partial or radical nephrectomy (SP-RAPN/SP-RARN) is relatively sparse. Kaouk et al. first reported three cases of SP-RAPN in patients with T1a renal cancer. All procedures were completed without conversion, and only one patient required embolization for acute bleeding, with no positive margins and preserved renal function in all cases [8].

Although the Da Vinci robot is the most successful and widely used surgical robot globally, its primary limitation is the lack of direct tactile feedback during procedures. The Da Vinci system relies on visual feedback, requiring surgeons to interpret visual cues in real time to judge the force exerted by instruments on tissues and assess tissue characteristics, which can affect surgical efficiency. The absence of tactile feedback may lead to excessive force on delicate tissues, resulting in organ damage, tissue tears, or suture breakage, compromising surgical safety. For example, excessive traction on the neurovascular bundle during RARP can cause nerve damage and delay the recovery of sexual function. However, the latest Da Vinci 5 system introduces notable advancements in robotic surgery, particularly through the integration of Force Feedback technology. This feature enables surgeons to feel the forces exerted on tissues, providing a tactile dimension that was previously absent. The system alerts surgeons when excessive pressure is applied, reducing the risk of tissue damage and enhancing surgical precision. This improvement is expected to decrease tissue damage by 43%, compared to earlier models. Additionally, the instrument sensors are able to generate post-operative, objective performance measurements on tissue for personal and training insights.

In summary, the Da Vinci SP system demonstrates feasibility and safety in urologic tumor surgery, representing a significant breakthrough in minimally invasive surgery. The system offers reduced trauma, less postoperative pain, and improved cosmetic outcomes. However, further validation through multi-center, large-sample randomized trials is necessary to confirm its clinical value.

### 2.2. Senhance System

The Senhance robot, developed by Asensus and approved by the FDA in 2017, is the first medical robot to offer a pressure-based tactile feedback system. This system allows surgeons to feel subtle pressure changes when the instruments contact tissues, enabling them to adjust their grip accordingly. Additionally, the Senhance system features eye-tracking technology for visual control. This function tracks eye movements by analyzing changes in the characteristics of the eyeball and surrounding tissues, as well as the angle of the iris. By autonomously projecting infrared beams onto the iris, the system extracts features to track real-time eye movements, determining the user’s needs and adjusting accordingly. This enables surgeons to control the endoscope’s view simply by moving their eyes, unlike the Da Vinci system, which requires foot pedals to adjust the endoscope’s perspective [9]. With the Senhance system, surgeons can control the endoscope’s view instantaneously by wearing special glasses paired with a 3D monitoring system. The Senhance robot has been reported in RARP and RARN surgeries, demonstrating good safety and feasibility [10,11].

### 2.3. Training and Learning Curve

The training and learning curve for these robotic systems can vary, depending on the surgeon’s prior experience with robotic surgery and the complexity of the procedures they intend to perform. For example. the training process for the Da Vinci SP system begins with didactic learning. Surgeons typically start with online modules or classroom sessions to understand the fundamentals of robotic surgery, the specific technology behind the Da Vinci SP, and its operational guidelines. The subsequent hands-on training involves practice with simulators or in lab settings where surgeons can work with cadavers or models to familiarize themselves with the unique SP design and the associated ergonomics, typically conducted in designated training centers or surgical labs. After that, surgeons typically perform their initial surgeries under the supervision of a proctor to ensure patient safety and surgical proficiency [12].

The learning curve is characterized by the period in which surgeons initially develop an improvement in performance over time, followed by a plateau phase, in which there is limited further improvement [13]. Surgeons with prior robotic experience typically experience a faster learning curve, whereas those without face a steeper learning curve. Learning curves typically use metrics like operative time, blood loss, complication rates, and procedure-specific outcomes as surrogates for technical performance. A systematic review demonstrated that the robot-assisted laparoscopic prostatectomy learning curve for operative time spanned 10 to 250 cases and the RAPN learning curve for warm ischemia time ranged from 4 to 150 cases [14]. For RARC, achieving proficiency in operative time and lymph node yield required between 10 and 50 cases [15]. This broad range complicates the use of learning curve data to guide training programs, due to significant inter-trainee differences. Furthermore, each system itself differs and transitioning between robotic systems, even for experienced surgeons, introduces new learning challenges.

### 2.4. Cost and Accessibility

The cost and accessibility of these cutting-edge robotic surgical systems pose significant challenges for widespread adoption, particularly in less-resourced healthcare settings. Initial acquisition cost of the Da Vinci SP system can range between USD 1.5 million and USD 2.5 million per unit [16]. In addition, the costs of maintenance, consumables, and training are expensive. A Swedish multi-center trial comparing RARP to radical retropubic prostatectomy estimated an increased cost of USD 3837 per robotic procedure with a two-year follow-up [17]. The high cost of these system limits their accessibility primarily to large, well-funded hospitals, particularly in the U.S., Europe, and parts of Asia [18]. Many smaller hospitals, particularly in rural areas or in lower-income countries, where traditional open or laparoscopic surgery remains the standard, may not be able to afford to acquire or maintain this technology. Thus, it is notable that patients who have access to these robotic systems tend to have higher socioeconomic status, which often correlates with better overall health, more comprehensive insurance coverage, and the ability to seek care at top-tier medical institutions. This creates a potential bias in outcomes, as these patients may already experience better health outcomes compared to the general population, independent of the technology itself.

While the costs of these systems are substantial, their potential to reduce overall healthcare costs is significant. The key benefit of robotic surgery is its potential to minimize patient length of stay (LOS). With more precise operations, reduced blood loss, and smaller incisions, patients often experience fewer complications and quicker recoveries, allowing for same-day discharge or shorter hospitalizations. In complex surgeries, this transformation of traditionally multi-day stays into outpatient or short-stay cases can result in significant cost savings for healthcare systems. Orecchia et al. pointed out the feasibility and safety of transitioning RARP to a day-case procedure to reduce the economic burden of the healthcare system [19]. Although robotic surgery involves high investment, its ability to shorten LOS and transform complex procedures into day cases could lead to long-term cost savings.

### 2.5. Other Robotic Systems

Several new robotic systems have recently entered the market, such as the Versius, Hugo, and Hinotori systems [20]. The Versius system features a versatile open surgeon console, allowing for both sitting and standing positions. Its compact design includes individual bedside units for each robotic arm, and it incorporates V-wrist technology, providing seven degrees of freedom (DoF) and 360-degree wrist motion to control 5 mm sterilizable instruments [21]. The Hugo system employs an open surgeon console with four individual arm carts and a system tower. Similar to the Senhance system, it offers a 3D surgical display that requires specialized glasses for viewing. The system uses an 11 mm port for the endoscopic camera and 8 mm ports for surgical instruments [22]. The Hinotori system is equipped with a four-arm operational unit and introduces an additional DoF compared to the da Vinci X/Xi systems, potentially enabling smoother and more refined movements [23]. The advent of multiple systems has significantly driven innovation, such as enhanced haptic feedback, improved camera systems, and more ergonomic designs. These improvements can lead to better surgical precision, which is crucial for urologic procedures involving delicate tissues and structures. Moreover, new systems often emphasize cost reduction and greater availability for a wider range of hospitals, including smaller institutions that previously could not afford robotic surgery platforms. For example, Versius and Hugo are marketed as more affordable alternatives, potentially reducing the economic barrier for institutions.

### 2.6. Comparison of Robotic Surgery versus Traditional Surgery

Robotic surgery has revolutionized the treatment of urologic tumors, providing notable perioperative and postoperative advantages over traditional open and laparoscopic surgical techniques [24]. Its precise control of instruments and the ability to identify small vessels enables reduced intraoperative blood loss. Studies, particularly in RARP and RAPN, consistently show a decreased need for blood transfusions compared to traditional approaches [25,26]. Consequently, robotic surgery leads to less postoperative pain, fewer postoperative complications, faster recovery times, and shorter hospital stays. Functional outcomes related to urinary continence and sexual function are critical for quality of life in men with prostate cancer. Evidence suggests that RARP offers better continence and erectile-function recovery compared to laparoscopic radical prostatectomy [27]. The precise movements allowed by robotic systems contribute to better nerve-sparing techniques, which are vital for preserving these functions. In RAPN, the ability to precisely excise tumors while preserving kidney function appears to be superior to traditional approaches [28]. In terms of oncological outcomes, prostate cancer patients undergoing RARP show lower rates of positive surgical margins and biochemical recurrence compared to those receiving open surgery [29]. RAPN demonstrates similar oncological outcomes to those of open partial nephrectomy for complex renal tumors, with no significant differences in positive margins, local recurrence, overall survival, and recurrence-free survival [30]. Furthermore, higher patient satisfaction and better overall quality of life were often reported in patients undergoing robotic surgery, due to quicker return to normal activities, better cosmetic outcomes, and effective tumor control [31].

Understanding the economic implications of robotic surgery is crucial for healthcare systems. Traditional surgery typically results in longer recovery times, due to larger incisions and more extensive tissue damage, whereas the minimally invasive nature of robotic surgery leads to fewer complications, faster recovery, and reduced hospital stays. This means fewer resources are spent on postoperative care, and hospitals can serve more patients in a given time period, improving the overall cost efficiency of healthcare delivery. Moreover, the reduced incidence of complications and reoperations contributes to long-term healthcare savings, making robotic surgery an economically attractive option. While traditional surgery may require a larger team to assist, robotic surgery typically needs smaller surgical teams and allows surgeons to perform more complex procedures with greater ease, which enhances the efficiency of healthcare personnel. Therefore, despite the high initial costs, the long-term savings generated by fewer complications, reduced hospital stays, and increased procedural efficiency often justify the investment. Over time, as technology becomes more widespread and competitive, the costs of robotic systems may decrease, making them more accessible in various economic settings.

## 3. Advances in Assistive Technologies

### 3.1. Three-Dimensional (3D) Reconstruction Combined with Augmented Reality (AR)

Preoperative planning is a crucial step in robotic surgery, ensuring precision and increasing the likelihood of success. With the rapid advancements in medical imaging and computer technologies, three-dimensional (3D) reconstruction and surgical navigation have become essential tools for preoperative planning. Three-dimensional reconstruction converts two-dimensional (2D) data from CT or MRI scans into 3D visualized digital models, providing a detailed view of the lesion [32]. Surgical navigation offers precise localization of the lesion, guiding the surgical plan and execution [33]. Augmented reality (AR) is an advanced technology that merges virtual information with the real world, enhancing sensory perception. AR features seamless integration of the virtual and real, accurate positioning, and real-time interaction, allowing surgeons to simulate and refine surgical plans through intuitive interaction [34]. Recently, combining 3D reconstruction with AR, where preoperative imaging-based 3D models are integrated with intraoperative anatomical data, has emerged as a novel approach in robotic surgery for urologic tumors.

Porpiglia et al. pioneered the use of these technologies together in RARP. They created high-resolution 3D models of the prostate and surrounding structures from multiparametric MRI scans and overlaid these models onto the Da Vinci robotic system’s stereoscopic surgical field, guiding the procedure effectively [35]. In a subsequent study, 19 patients with suspected extracapsular extension (ECE) of prostate cancer based on preoperative MRI were treated, and 15 (78.95%) were confirmed postoperatively as stage T3a, indicating the presence of ECE. Among these 15 patients, selective biopsy guided by 3D reconstruction combined with AR detected cancer cells near the neurovascular bundle adjacent to the extracapsular lesion in 11 cases (73.33%), suggesting that this approach may enhance the accuracy of ECE detection and optimize surgical clearance [36]. In radical prostatectomy, preserving nerves is critical to reducing the adverse impact on sexual function. However, during nerve-sparing procedures, the prostate may deform due to traction by the robotic arms, causing misalignment with static virtual images. To address this, the team developed a 3D elastic reconstruction that simulates prostate deformation using nonlinear parameters and assessed its accuracy in identifying extracapsular involvement in combination with AR [37]. The 3D elastic model outperformed traditional 2D MRI in targeting involved capsule locations during nerve-sparing surgery (100% vs. 47%, *p* < 0.05). Given the potential for prostate cancer’s primary lesions to metastasize, leading to ECE, lymph node metastasis, or recurrence, accurate detection of these lesions is vital. Bianchi et al. utilized 3D reconstruction combined with AR to identify primary lesions in real-time during RARP and performed frozen section analysis of surrounding tissues [38]. The group using 3D reconstruction combined with AR had a significantly lower rate of positive surgical margins compared to those using multiparametric MRI alone (5% vs. 20%, *p* = 0.01).

Porpiglia et al. also presented their experience using intraoperative navigation with 3D reconstruction combined with AR during complex robotic partial nephrectomy for renal tumors [39]. By integrating the virtual 3D kidney model with the stereoscopic surgical field, they achieved real-time surgical guidance. Adjusting the transparency of the 3D model allowed surgeons to identify endophytic tumors within the renal parenchyma and precisely locate surrounding vessels and the collecting system. For posterior renal tumors, where significant mobilization and rotation of the kidney are required, static virtual images often fail to align accurately with the real-time anatomy. However, the 3D elastic reconstruction combined with AR enabled real-time adjustments to the virtual kidney’s position, ensuring accurate alignment with the actual organ and enhancing navigation. Compared to conventional ultrasound navigation, 3D reconstruction combined with AR resulted in a lower rate of total renal artery clamping, reduced damage to the collecting system, and improved tumor enucleation rates. Postoperative complications were fewer, and the degree of reduced renal blood flow was less pronounced, suggesting better preservation of normal renal tissue and enhanced postoperative recovery.

The combination of 3D reconstruction and AR in robot-assisted surgery for urologic tumors offers significant advantages. Preoperatively, AR provides an immersive environment for optimizing surgical plans, while intraoperatively, it allows precise real-time visualization of the tumor’s relationship with surrounding vessels, nerves, or critical organs, improving anatomical localization and reducing severe complications. However, there are limitations, such as the dependence on multiparametric MRI for accurate 3D reconstruction, potential misalignment of virtual models with the surgical field, and issues with navigation precision. Despite these challenges, ongoing technological improvements suggest substantial potential for further development and application in this field.

### 3.2. Indocyanine Green (ICG) Fluorescence Imaging Technology

Indocyanine green (ICG) is a water-soluble fluorescent dye that emits green fluorescence when excited by near-infrared light. ICG can bind to bilirubin translocase, which is secreted by renal proximal tubules, causing normal renal tissue to exhibit isofluorescence. In contrast, renal cancer cells secrete less of this enzyme, resulting in weaker fluorescence, thereby aiding in the differentiation of tumor tissue from surrounding normal tissue. Additionally, ICG can bind to plasma proteins, causing well-perfused renal tissue to appear with high fluorescence, while ischemic renal tissue appears as dark areas with weak fluorescence, providing a clear visualization of renal vasculature and tissue perfusion [40].

During RAPN, ischemia–reperfusion injury caused by clamping the main renal artery can lead to renal function impairment. Selective clamping of renal artery branches, however, can reduce the extent of ischemia and minimize renal damage. ICG-guided RAPN allows for real-time fluorescence imaging of renal parenchymal perfusion, enabling precise assessment of the ischemic area corresponding to the clamped renal artery branch and its alignment with the tumor. If the ischemic area matches the tumor region, precise excision can be performed. If not, a sliding suture technique can be used to minimize ischemia time [41]. A large multicenter study found that among 318 patients undergoing ICG-guided RAPN, 254 patients (79.9%) achieved the “trifecta” outcomes (no Clavien–Dindo grade II or higher complications, warm ischemia time <25 min, and negative surgical margins) [42]. The study demonstrated that ICG is particularly useful for intraoperative guidance in complex renal tumors with challenging vascular anatomy. A meta-analysis that included six studies with a total of 369 patients compared short-term outcomes between ICG-guided RAPN and standard RAPN. The ICG-guided group showed higher glomerular filtration rates and better ipsilateral renal function, indicating that selective clamping of renal artery branches under ICG guidance results in better functional outcomes in the short term [43].

In RARP with lymph node dissection, ICG is injected transperineally into the prostate, allowing real-time observation of fluorescence distribution from the prostate to pelvic lymph nodes. Surgeons can then excise the highlighted nodular and tubular structures [44]. Studies have reported that ICG injection into the prostate during extended lymphadenectomy can identify sentinel lymph nodes in 76% of patients, with a sensitivity of 100% and a specificity of 75.4% [45]. Additionally, intraoperative intravenous injection of ICG can delineate the major arteries within the neurovascular bundles, assisting in nerve-sparing decisions, which may enhance postoperative recovery of urinary continence and sexual function [46].

## 4. Advances in Artificial Intelligence (AI)

Artificial intelligence (AI) is a rapidly evolving field focused on the development of systems that simulate, extend, and augment human intelligence, encompassing principles, thought processes, and specialized skills [47]. AI aims to identify patterns and connections within vast datasets, enabling the creation of intelligent machines capable of responding in ways that resemble human intelligence. In robotic surgery, the extensive data generated, such as intraoperative video recordings, dynamics of surgical instrument manipulation, preoperative laboratory and imaging data, postoperative complications, and functional and oncological outcomes, can be deeply analyzed by AI. Machine learning applied to these datasets can be utilized for evaluating surgical performance, simulating procedures, and assisting in preoperative planning, intraoperative decision-making, and postoperative outcome prediction. Current research is mostly focused on using deep learning to predict postoperative outcomes. For instance, Hung et al. prospectively collected data on surgical instrument trajectories (including movement time, distance traveled, and wrist angles), endoscope movement, and energy usage during RARP. From these data, they derived 41 automated performance metrics, which, when combined with 16 clinical and pathological indicators, were used to predict postoperative urinary continence recovery with high accuracy using deep learning techniques [48].

During vesico-urethral anastomosis (VUA) in RARP, the surgeon’s choice of suturing technique significantly impacts patient outcomes. Luongo et al. utilized computer vision and deep learning to automatically identify suturing maneuvers in 2395 surgical videos, achieving an accuracy of 79% in 511 validation videos [49]. Their study demonstrated that computer vision can not only recognize suturing actions, but can also distinguish subtle differences in suturing techniques, laying the foundation for future robotic platforms capable of autonomous suturing. Zia et al. developed a novel model based on convolutional neural networks (CNNs) and long short-term memory (LSTM) networks to identify and quantify the surgical process of RARP into 12 steps by analyzing surgical videos [50]. Similarly, Nakawala et al. used CNN and LSTM to identify the surgical workflow in RAPN, confirming the current surgical step, predicting the next step, and inferring surgical phases and actions based on intraoperative anatomical information [51]. These studies aim to automate the recognition and quantification of surgical procedures, advancing the field of robotic surgery automation.

The ultimate goal of AI development in robotic surgery is full automation, but the current level of automation in medical robots is still in its infancy [52]. The degree of automation is classified into six levels. Level 0 denotes no automation, with the robot entirely controlled by the surgeon. Level 1 is robotic assistance, where the surgeon maintains continuous control of the system, and the robot provides mechanical guidance and assistance. Level 2 is task automation, where the surgeon intermittently controls the system, and the robot autonomously performs specific tasks. Level 3 is conditional automation, where the robot generates task strategies that require human selection among them, allowing the robot to complete tasks without human intervention. Level 4 is high automation, where the robot makes medical decisions and performs surgery under the surgeon’s supervision. Level 5 represents full automation, where the robot independently performs the entire surgery. The widely used Da Vinci surgical robot currently operates at Level 0 automation. Automated robots are trained through explicit learning via direct programming or implicit learning by observing surgeons or watching videos [53]. An example of explicit learning is the Smart Tissue Autonomous Robot (STAR). Researchers programmed STAR to autonomously perform suturing on pig intestines using its vision, tools, and computational abilities [54]. About 40% of the procedure involved guidance from researchers, while the remaining 60% was completed autonomously by STAR, categorizing it as Level 2 automation. STAR’s suturing was found to be more precise and less prone to leakage, compared to experienced surgeons. Implicit learning, also known as imitation learning, involves breaking down a surgical task into subtasks, followed by recognition and modeling of these subtasks for execution. Recent research has shown that the Da Vinci robot, after watching numerous videos, can imitate suturing, representing a form of implicit learning [55].

However, fully automated robotic surgery is still in its early stages. Robust data support is essential for achieving automation, while advanced computational algorithms and technologies are the tools that will drive its development. It is anticipated that fully automated robots will eventually be developed, capable of formulating optimal surgical plans based on disease-related parameters and big data, performing autonomous surgery without the need for human supervision, and correcting errors in real time, significantly enhancing surgical quality and efficiency.

Still, fully automated robotic surgery systems present a range of ethical and regulatory challenges that require careful consideration. One of the primary concerns is patient safety and accountability. While automation has the potential to reduce human error, the unpredictability of machine learning algorithms in complex, real-time environments like anatomical variation raises concerns about malfunction or unintended outcomes. If an autonomous system makes a critical error, accountability becomes murky. Determining who is responsible—AI developers, hospitals, or the manufacturer—poses a significant ethical dilemma. This concern is further complicated by the opaque nature of AI decision-making processes, often referred to as the “black box” problem, where it is difficult to trace how a system arrived at a specific decision during surgery. Regulatory frameworks, which are traditionally designed for human-controlled medical devices, also face challenges in accommodating fully autonomous systems. Existing regulations, such as those from the FDA or CE marking, are not yet fully equipped to evaluate the nuances of machine-driven decisions in a surgical setting. To address these gaps, regulatory bodies must develop new standards for testing, validating, and monitoring these systems over time, ensuring that they meet the highest safety and ethical standards. Moreover, there are concerns regarding data privacy, as automated systems often rely on vast numbers of patient data to function and improve. Ensuring that these data are securely handled and that patients retain control over their personal health information is critical in preserving trust in these technologies.

## 5. Conclusions

Over the past two decades, robotic surgery has become increasingly mature in the treatment of urologic tumors. It has addressed many limitations of laparoscopic surgery, ensuring surgical safety while achieving oncological and functional outcomes that are comparable to, or even better than, those of traditional laparoscopic procedures. Robotic surgery represents a significant trend in the evolution of urologic surgery. Recent advancements include the Da Vinci SP system’s single-port mechanical arm, which minimizes surgical trauma; the Senhance system’s unique tactile feedback and eye-tracking, which offer a more immersive experience for the surgeon; and the integration of three-dimensional reconstruction with augmented reality technology, as well as intraoperative imaging techniques, which enhance surgical precision. Moreover, the ongoing development of artificial intelligence is poised to elevate the application of robotic surgery to new heights. With continuous innovation and refinement, robotic surgery is expected to advance toward minimal invasiveness and greater precision and intelligence.

## Data Availability

Not applicable.
